# *In vivo* evaluation of homeostatic effects of *Echis carinatus* snake venom in Iran

**DOI:** 10.1186/1678-9199-19-3

**Published:** 2013-02-27

**Authors:** Hossein Salmanizadeh, Mahdi Babaie, Hossein Zolfagharian

**Affiliations:** 1 Young Researchers and Elites club, Science & Research Branch, Islamic Azad University, Tehran, Iran; 2 Department of Venomous Animals and Antivenom Production, Razi Vaccine and Serum Research Institute, Karaj, Iran

**Keywords:** Snake venom, Procoagulant activity, Blood coagulation, *Echis carinatus*, Chromatography, LD_50_

## Abstract

**Background:**

The venom of the family Viperidae, including the saw-scaled viper, is rich in serine proteinases and metalloproteinases, which affect the nervous system, complementary system, blood coagulation, platelet aggregation and blood pressure. One of the most prominent effects of the snake venom of *Echis carinatus* (Ec) is its coagulation activity, used for killing prey.

**Materials and methods:**

Subfractions F1A and F1B were isolated from Ec crude venom by a combination of gel chromatography (Sephadex G-75) and ion exchange chromatography on a DEAE-Sepharose (DE-52). These subfractions were then intravenously (IV) injected into NIH male mice. Blood samples were taken before and after the administration of these subfractions. Times for prothrombin, partial thromboplastin and fibrinogen were recorded.

**Results and conclusions:**

Comparison of the prothrombin time before and after F1A and F1B administrations showed that time for blood coagulation after injection is shorter than that of normal blood coagulation and also reduced coagulation time after Ec crude venom injection. This difference in coagulation time shows the intense coagulation activity of these subfractions that significantly increase the coagulation cascade rate and Causes to quick blood coagulation. The LD50 of the Ec crude venom was also determined to be 11.1 μg/mouse. Different crude venom doses were prepared with physiological serum and injected into four mice. Comparison of the prothrombin times after injection of subfractions F1A and F1B showed that the rate of mouse blood coagulation increases considerably. Comparing the partial thromboplastin times after injecting these subfractions with this normal test time showed that the activity rate of intrinsic blood coagulation system rose sharply in mice. Finally, by comparing the fibrinogen time after subfraction injections and normal test time, we can infer intense activation of coagulation cascade and fibrin production.

## Background

Snakebite affects around 2.5 million humans annually, accounting for more than 100,000 deaths. Coagulopathy is a significant cause of both morbidity and mortality in these patients, either directly, or indirectly. Clinical concerns in snakebite include types of coagulopathy (procoagulant, anticoagulant, fibrinogen clotting, fibrinolytic, platelet-active, thrombotic, hemorrhagic), diagnosis and treatment [1-3].

Where available, antivenom is the most effective treatment against snakebites, while standard treatments for other forms of coagulopathy, such as factor replacement therapy and heparin, are either ineffective or dangerous, except in specific situations. Interference with aspects of the human hemostatic system is a common theme that encompasses all four families of venomous snakes including *Echis carinatus* (from Viperidae family) in Iran [4].

Among the many potential effects of envenoming by snakes in humans, only a few broad categories are of major clinical significance including paralysis and mild stroke; systemic myolysis; coagulopathy and hemorrhage; renal damage and failure; cardiotoxicity; and local tissue injury at the bite site [4,5].

Any single snake species may possess toxins that act in one or more of these categories, though rarely all six. In the past, it was wrongly assumed that a single ophidian species would generally cause either local or systemic effects and that vipers caused local and/or hemorrhagic effects, while elapids caused purely systemic, non-hemorrhagic effects [6-9].

In this research, the effects of *Echis carinatus* crude venom and its fractions on mice were analyzed. Moreover, the results of coagulation tests on its venom were recorded.

## Methods

### Materials

Sephadex G-75, DEAE-Sepharose, was purchased from Pharmacia (Sweden). CaCl_2_, thromboplastin-D and APTT-XL kits were purchased from Fisher Diagnostics (Germany). The other reagents and chemicals of analytical grade were purchased from Fluka and Merck.

### Venom and animals

Sixty milligrams of *Echis carinatus* venom was obtained from the Venomous Animal Unit of Razi Vaccine and Serum Research Institute, Iran. Fifty-two NIH mice were supplied from the Laboratory Animal Breeding Unit of Razi Vaccine and Serum Research Institute, Iran. Mouse blood samples were centrifuged for ten minutes at 3,000 rpm. The plasma obtained was used for the prothrombin time (PT), partial thromboplastin time (PTT) and fibrinogen time (FT) tests.

### Prothrombin time (PT) test

The thromboplastin-D vial was brought to the laboratory and equilibrated to room temperature. Two hundred microliters of the solution was poured into a hemolysis tube, and incubated for three minutes at 37°C. One hundred microliters of mouse plasma was poured into a hemolysis tube containing 200 μL of thromboplastin-D solution at the same moment that the chronometer was switched on. A glass pipe containing 200 μL of thromboplastin-D solution and 100 μL of the plasma was incubated for five minutes at 37°C. The process of plasma clotting was observed and the time recorded [10,11].

### Partial thromboplastin time (APTT) test

The APTT-XL solution vial was equilibrated to room temperature in the laboratory. One hundred microliters of this solution was then poured into a hemolysis pipe; 100 μL of mouse plasma was added to it and the mixture was incubated for three minutes at 37°C. Subsequently, 100 μL of CaCl_2_ was added and the chronometer was simultaneously switched on. The preparation was shaken for 19 s in *bain-marie* (water at 37°C). The process of plasma clotting was observed and the time recorded [11].

### Fibrinogen time (FT) test

Half an hour before conducting the test, the reagents were taken out of the refrigerator in order to equilibrate their temperature to room temperature. First step, dilution: 0.1 mL of plasma was diluted with 0.9 mL of the test kit diluting buffer to achieve the plasma dilution 1:10. Incubation: 0.2 mL of the diluted plasma was poured into a hemolysis pipe for incubation for two minutes at 37°C. Clot formation: the thrombin containing reagent should have the lab temperature (25°C) throughout the test time. It should never be incubated at 37°C. Two minutes after incubation, 0.1 mL of the thrombin containing reagent was added to the diluted plasma and the chronometer simultaneously switched on. As soon as the first signs of clotting were observed, time was recorded and the fibrinogen level determined [12,13].

### Measurement of the Ec crude venom coagulation activity

For measuring the Iranain *Echis carinatus* crude venom coagulation activity, 10 mg of the crude venom was initially used to prepare different concentrations (1, 0.1, 0.01 mg/mL). These concentrations were them exposed in the PT test.

### Isolation and purification of coagulation factors

Isolation and purification of coagulation factors were performed using 50 mg of Ec crude venom using a combination of gel chromatography and ion exchange chromatography. Ec crude venom was primarily isolated using gel chromatography (Sephadex G-75) column which initially gained equilibrium using 20 mM ammonium acetate buffer (pH 6.8). That is, the column input and output pH became the same. Fifty milligrams of Ec crude venom was dissolved in 4 mL of ammonium acetate buffer. The solution was then centrifuged for 15 min at 4°C at 14,000 rpm. The supernatant was isolated and gradually poured into the gel chromatography Sephadex G-75 (200 × 3 cm) column using a special syringe. The sample was then well absorbed by the column and was automatically eluted with ammonium acetate buffer using an automatic collector at the flow rate of 60 mL/h for 24 h. The absorption of the resulting solution was read using a spectrophotometer at 280 nm and relevant absorption curve was drawn in terms of the tube number [10,14,15].

For taking the ammonium acetate buffer out of the solutions, each of the peaks was dialyzed for 24 h with distilled water. After dialysis, the fractions were concentrated at 4°C with sucrose. The ion exchange chromatography (1.5 × 25 cm) column was equilibrated with Tris–HCl 0.05 mM buffer (pH 8.2), i.e., the input buffer was the same as the output buffer. For the peaks obtained by gel chromatography, the fraction that exhibited coagulation activity was exposed to ion exchange chromatography for further isolation and subfractionation. Initially, a certain amount of the chromatography first peak gradually entered the column (4°C) which was then eluted with Tris–HCl 0.05 mM buffer. Subsequently, the column was eluted with Tris–HCl 0.05 mM buffer and gradient buffer; pH 8.2 (Nacl concentration 0.1 to 0.4 mM). The ion exchange chromatography output solution was collected by an automatic collector at a flow rate of 20 mL/h for 24 h. The absorption of collected tubes was read using a spectrophotometer at 280 nm and relevant optical absorption curve was drawn in terms of the tube number [10,14-16]. The subfractions were pooled and dialyzed like in gel chromatography.

### Estimation of lethal dose venom (determination of LD_50_)

This test was conducted according to the method by Meier and Theakston [17]. Different doses of crude venom were prepared in physiological serum and were each injected into four mice (2 mL/dose, 0.5 mL/mouse). The doses were chosen was so that no mouse would die at the lower dose, and all mice would die at the higher dose. Mouse mortality within 24 h was recorded and each sample LD_50_ was calculated. Upon recording of mortality, the Spearman-Karber statistical method was used for LD_50_ calculation [16,18,19].

## Results

*Echis carinatus* crude venom decreases coagulation time of mouse plasma in relation to its normal levels. Thus, the venom shows coagulation properties. Based on the results of Table 1, it is clear that all Ec venom concentrations have coagulation properties. Therefore, as the venom concentration increases, its coagulation properties will also augment. The existence of coagulation factors in Ec venom was then established.

**Table 1:** Prothrombin time test for different concentrations of crude venom of Ec

**Preamble**	**SD**	**p value**	**Mean (s)**							**Concentration of venom (mg/mL)**
Formed fibrin fibers	1.634	0.025, < 0.05	20.9	22.4	19.1	22.1	22.6	19.2	20.1	0.01
Increased size of clot	0.794	0.024, < 0.05	12.1	12.4	11.6	12	13	11	13	0.1
Formed complete clot	0.640	0.025, < 0.05	8.6	9.2	8.4	8	9.5	9	8	1
Formed complete clot	0.592	–	13.2	12.7	13.2	12.5	14	13.4	13.8	Instance

### Ec crude venom Gel chromatography

**Figure 1:**
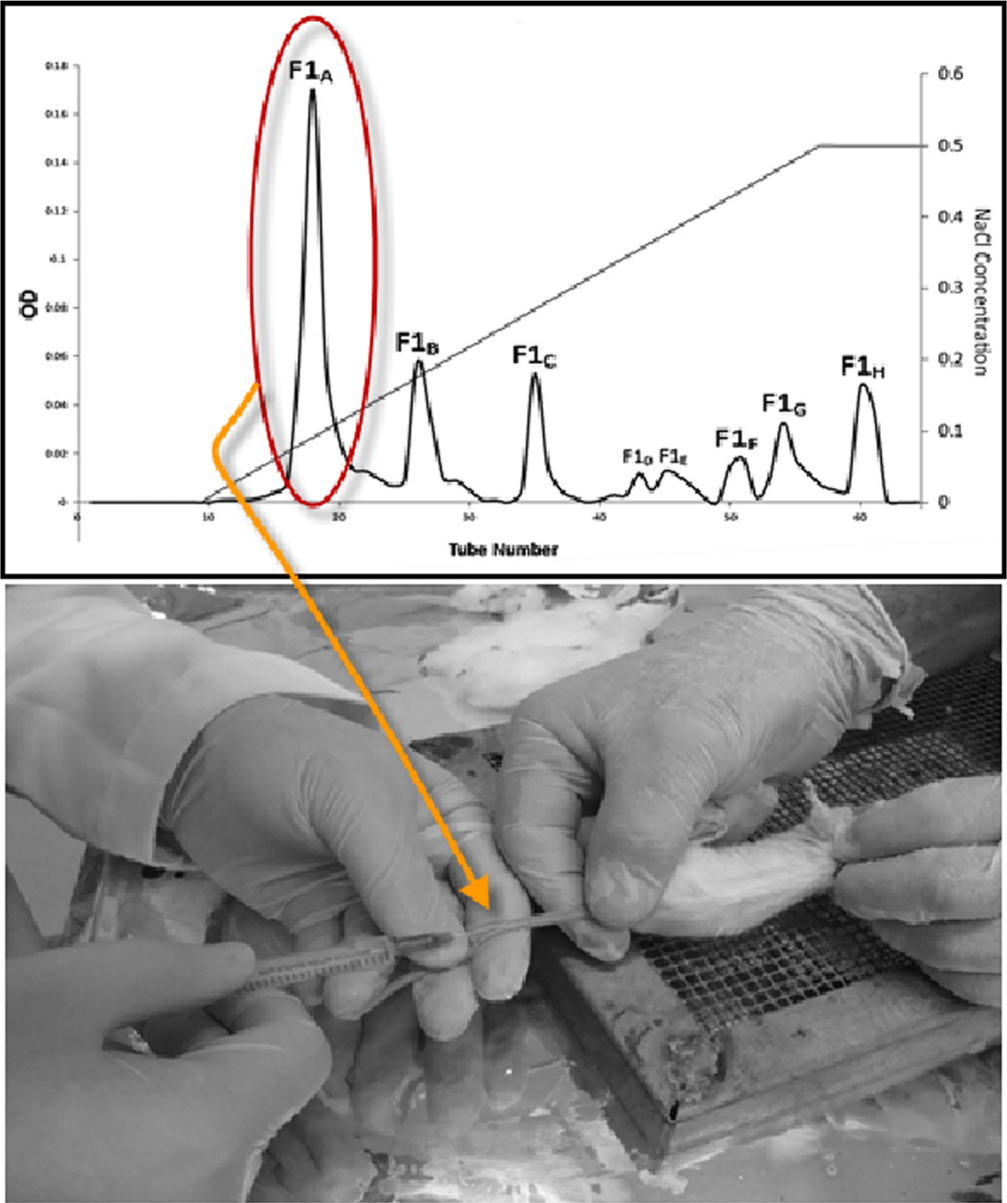
**Gel chromatography (Sephadex G-75) of 50 mg of *Echis carinatus *** **crude venom.**

By performing gel chromatography, five fractions were obtained according to Figure 1, respectively labeled F_1_ to F_5_. As per the existing standards on gel chromatography in which protein molecules separate by size; larger molecules pass more freely, appearing in the earlier fractions, F_1_ was considered the peak with the highest amount of protein. Regarding the gel chromatography isolation process based on molecular weight, peaks or fractions respectively containing less total protein will exit from the gel chromatography column. Fractions F_2_ to F_5_ contain proteins with molecular weights lower than that of F_1_.

### Study of the coagulation activity of the fractions from Gel chromatography

Regarding Table 2, by conducting the PT test on mouse plasma, it was shown that fraction F_1_ diminished the coagulation time and that other fractions increased it.

**Table 2:** Prothrombin time test on mouse plasma by using fractions obtained from gel chromatography

**Mean**	**PT sample 6 (s)**	**PT sample 5 (s)**	**PT sample 4 (s)**	**PT sample 3 (s)**	**PT sample 2 (s)**	**PT sample 1 (s)**	**Fraction**
17.08	16.7	17.3	16.8	17.4	16.5	17.8	F_1_
35.4	37.4	33.9	35	36.5	32	38	F_2_
–	More than 5 min	More than 5 min	More than 5 min	More than 5 min	More than 5 min	More than 5 min	F_3_
–	More than 5 min	More than 5 min	More than 5 min	More than 5 min	More than 5 min	More than 5 min	F_4_
–	More than 5 min	More than 5 min	More than 5 min	More than 5 min	More than 5 min	More than 5 min	F_5_

### Isolation of subfractions F_1_ using Ion exchange chromatography

**Figure 2:**
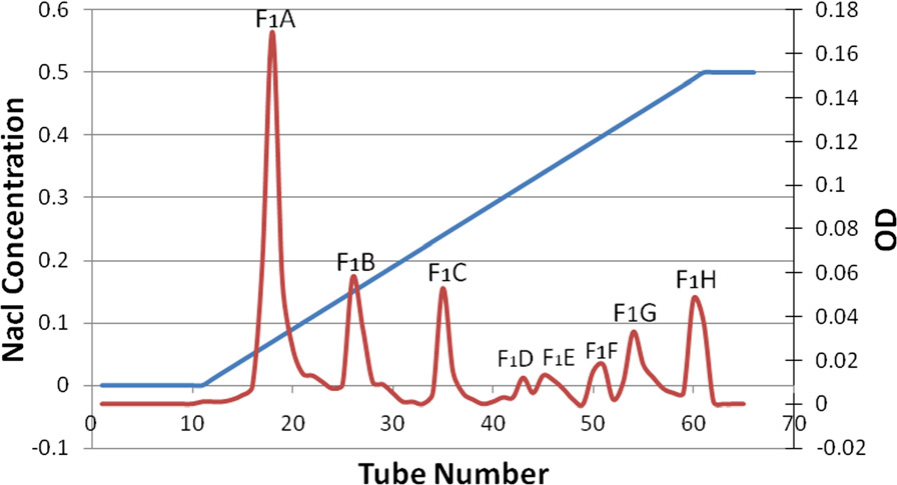
**Ion exchange chromatography of F**_**1 **_**fraction.**

Among the fractions obtained from gel chromatography fraction F_1_ was selected for furhter isolation because of its lower coagulation time, and was taken to the DEAE-Sepharose ion exchange column. The eight fractions obtained were thus labeled F_1_A to F_1_H (Figure 2).

### Study of the F_1_A and F_1_B subfractions coagulation activity

The PT test was frequently conducted on human plasma using subfractions F_1_A and F_1_B. These results (p ≤ 0.05) showed a significantly more powerful coagulation activity of these subfractions when compared with others. The mean PT obtained for subfractions F_1_A was 7 s and for subfractions F_1_B, 5 s. Compared with the normal time, this interval is lower, showing the intense coagulation properties of these subfractions. For more investigation into the coagulation activity, these subfractions were selected for injection into mice.

### Injection of subfractions F_1_A and F_1_B

Subfractions F_1_A and F_1_B were intravenously (IV) injected into six NIH mice. Tables 3 and 4 show the results of the PT, PTT and FT tests before and after injection.

**Table 3:** **Results of PT, PTT and FT tests before and after the injection of F**_**1**_**A subfraction**

**FT after injection (s)**	**FT before injection (s)**	**PTT after injection (s)**	**PTT before injection (s)**	**PT after injection (s)**	**PT before injection (s)**	**Number of mice**	
47	25.2	48	30	6	11	1	Injection F_1_A subfraction with 10 μg/mL concentration
33	22	51	32	5	13.6	2	
50	19	52	37	7	12.2	3	
42	22	49	33.4	8	14.1	4	
49	23.1	50.5	34.2	9	13.9	5	
37	24.2	53	33	6	12	6	
43	22.5	50.5	33.2	6.8	12.8	Mean	

**Table 4:** **Results of PT, PTT and FT tests before and after the injection of F**_**1**_**B subfraction**

**FT after injection (s)**	**FT before injection (s)**	**PTT after injection (s)**	**PTT before injection (s)**	**PT after injection (s)**	**PT before injection (s)**	**Number of mice**	
47	22.1	50	29	4	11.2	1	Injection F_1_B subfraction with 10 μg/mL concentration
39	26	50.5	37	3.3	12.6	2	
41	24	49.5	39	4	11.8	3	
43	23.2	48.8	30	4	14.1	4	
45.1	21	52	29.2	5	13.3	5	
46	19	51	31.2	3	13	6	
43.5	22.55	50.3	32.5	3.8	12.6	Mean	

### Estimation of LD_50_

The Spearman-Karber statistical method was used to calculate LD_50_ as follows:

(1) $$\begin{array}{l} {\text{M}=\text{X}}_{100}\text{±d/n}\left(\mathrm{\Sigma r}-\text{n}/2\right)\\ {\text{M}=\mathrm{Log}\;\mathrm{LD}}_{50}\\ {\text{X}}_{100}=\;\mathrm{Log}17.5\;=\;1.24\\ \text{n}\;=\;4\\ \text{t}0.05\;=\;2.20\\ \left(\mathrm{For}\;4+4+-1\;\mathrm{degrees}\;\mathrm{of}\;\mathrm{freedom}\right)\\ \text{M}\;=\;1.24\;\pm 0.097/4\;\left(0+1+0+0+3+2+4–4/2\right)\\ \text{M}\;=\;1.24\;\pm 0.194\\ \text{M}\;=\;1.046\\ {\mathrm{LD}}_{50}=\;\mathrm{Antilog}\;1.046\\ {\mathrm{LD}}_{50}=\;11.1\mu \text{g}\\ \left[\mathrm{Antilog}\;\left(1,24+0,\mathrm{and}\;194\right)\;\mathrm{is}\;\mathrm{not}\;a\;\mathrm{proper}\;\mathrm{number}\;\mathrm{and}\;\mathrm{will}\;\mathrm{thus}\;\mathrm{be}\;\mathrm{rejected}\right] \end{array}$$

Determination of the LD_50_ range:

(2) $$\begin{array}{l} \mathrm{Vm}\;=0.097^{2}/4^{2}\left(4–1\right)\;\left(0\times 4+1\times 3+0\times 4+0\times 4+0\times 4+3\times 1+2\times 2+4\times 0\right)\\ \mathrm{Vm}\;=0.00196\\ \mathrm{Antilog}\;\left(1.046\pm 2.20\surd 0.00196\right)=\;\mathrm{Antilog}\;\left(1.046+0.097\right)\\ \left(\mathrm{That}\;\mathrm{is}\;8.89\;\mu g\;\mathrm{and}\;13.89\;\mu g\right)\\ {\mathrm{LD}}_{50}=\;11.1\;\mu \text{g}/\mathrm{mouse}\;\left(8.89–13.89\right) \end{array}$$

## Discussion

It has been important for scientists to identify and study the compounds in snake venom. Nowadays, there are different manners to isolate and purify snake venom enzymes and proteins and study their effects. Chromatography is the most commonly used method for crude venom isolation.

*Echis carinatus* crude venom fractions isolated by chromatography showed that this method is useful for fraction separation. Viperidae venoms, including that of Ec, are rich in compounds that may be useful for medicine and pharmaceutics [20]. For measuring and confirming Ec crude venom coagulation activity, the PT test was conducted with different venom concentrations (Table 1). At lower concentrations, small clots are formed and coagulation time is longer, whereas at higher concentrations, larger clots are found and coagulation time is shorter [21].

A mean time of 8 s was obtained on the PT test conducted on mouse plasma with venom concentration at 1 mg/mL. When compared with normal PT (13.2 s), it is observed that crude venom at this concentration made the blood coagulation cascade more active and faster. If the normal PT is equal to 13.2 s, the rate of the coagulation cascade activity will become 100%, with its international normalized ratio (INR) equaling 1 (Table 1) [22].

For isolation, identification and investigation of the properties of Ec crude venom coagulation factors, a combination of gel chromatography and ion exchange chromatography was employed. Fifty milligrams of crude venom were subjected to gel chromatography (Sephadex G-75) and five fractions were obtained (F_1_ to F_5_). The isolation of subfractions was performed according to gel chromatography standards based on molecular weight. F_1_ showed the highest level of proteins among the fractions. Therefore, the total protein level also decreased from peak 2 to peak 5 (Figure 1). After gel chromatography, the PT test was conducted to specify coagulation and anticoagulation properties of each fraction (Table 2). The total time of PT was obtained for fraction F_1_, with a mean of 17.08 s and its coagulation cascade activity was equal to 58.8% and INR to 1.5. Coagulation tests were performed with fraction F_1_ and the coagulation cascade decreased, which could be due to venom toxic properties on the hemostatic system.

PT test showed that F_1_ was a coagulation fraction whereas other fractions were considered to be anticoagulation fractions. Then, fraction F_1_ was subjected to ion exchange chromatography (Figure 2). F_1_ ion exchange chromatography led to the formation of eight subfractions (F_1_A to F_1_H). The PT test was also conducted on mouse plasma using these subfractions. Regarding the PT test results, subfractions F_1_A (mean 6.8 s) and F_1_B (mean 3.8 s) were considered major coagulation fractions. Table 2 displays that the PT test using these subfractions drastically increased the coagulation cascade activity level, extending it to over 100%. Thus, they were selected for injection into mice.

Another study, similar to ours, was conducted on snake venoms. Joseph *et al.*[23] succeeded in purifying a prothrombin activator from *Tropidechis carinatus* venom using a combination of gel chromatography, ion exchange and HPLC methods. The purification phases were similar to our work.

A proteinase from *Vipera lebetina* snake venom, VLH_2_, has been similarly isolated using a combination of gel chromatography with Sephadex G-75 followed by ion exchange chromatography with Sepharose DEAE A-50 [24]. In another work, *Agkistrodon acutus* snake venom was exposed to ion exchange chromatography with Sepharose DEAE followed by gel chromatography on Sephacryl S-200 to isolate fractions with coagulation activities [25].

In our research, to further study in vivo the coagulation properties of these two subfractions, F_1_A and F_1_B were administered to male NIH mice. F_1_A was IV injected into six mice (concentration of 10 μg/mL), and F_1_B into other six animals. The mean PT before the F_1_A injection was 12.8 s which, according to Table 3, displays 100% activity of coagulation cascade and INR = 1. PTT and FT before F_1_A injection were also recorded. The mean PTT before administration was 31.7 s and the mean FT was 22.5 s.

About an hour after injection, blood samples were collected. The mean PT test after the F_1_A injection was 6.8 s, which enhanced the coagulation cascade more than 100% (Table 3). This rapid response of the coagulation cascade occurs in the animal body and generates clinical effects such as coagulopathy, which may provoke death. The mean PTT after injection was equal to 44.8 s. This value compared with the one before injection (31.7 s) is increased. With F_1_A, coagulation occurs without addition of the last test component, CaCl_2_ (Table 3).

The mean FT before injection was 22.5 s and after injection was 43 s. This difference in coagulation time can be indirectly attributed to failure and dysfunction in blood coagulation factors by the presence of procoagulation compounds like prothrombin activators. Moreover, subfraction F_1_B was IV injected into six mice at a concentration of 10 μg/mL (10 μg/mouse). Mouse plasma PT, PTT and FT were determined before injection. The mean PT before injection was 12.6 s, and the coagulation cascade was 100% with INR = 1 (Table 4).

An hour after the injection of F_1_B, blood samples were collected. The mean PT after injection was 3.8 s, which shows an intense activity of the coagulation cascade (more than 100%), as seen in Table 4. The mean PTT before injection was 32.5 s and 42.9 s after injection, indicating an increase in PTT. After injection of the subfraction F_1_B, similarly with F_1_A, plasma coagulation occurred without addition of the last component, CaCl_2_ (Table 4). The mean FT before injection was 22.55 s and 43.5 s after injection.

This coagulation difference may be indirectly attributed to failure and dysfunction in blood coagulation factors by the presence of procoagulation compounds, such as prothrombin activators. Statistical results suggest that H_0_ is rejected by both subfractions F_1_A and F_1_B and, hypothetically, H_1_ is accepted by both. The p value will thus be significant, p ≤ 0.05. In other words, according to H_1_, we will have Mμ_1_ – Mμ_2_ = 0. Other similar studies have also been conducted. For example, Gao *et al.*[26] fractionated the snake venom of *Micropechis ikaheka* into a few protein peaks with a combination of gel chromatography and ion exchange chromatography and tested relevant effects on blood coagulation. Their results corroborate ours concerning blood coagulation and anticoagulation factors [26].

*Halys agkistrodon* snake venom was analyzed by Ghorbanpur *et al.*[27] though gel chromatography. The crude venom was separated into five fractions (AH_1_-AH_5_), all of which were exposed to the PT test in order to study the coagulation process. Then, fraction AH_1_ was positive in terms of coagulation. The PT assessment method showed that AH_1_ had coagulation activities. Further purification steps were performed by ion exchange chromatography, generating five fractions (AH_11_-AH_15_), of which AH_14_ showed coagulation properties [27].

In 2000 and 2003, Oyama and Takahashi [28,29] succeeded in purifying a thrombin-like enzyme from *Trimeresurus elegans* snake venom in a three-phase method consisting of gel chromatography and two phases of ion exchange chromatography. This enzyme did not influence human fibrinogen. However, it showed coagulation effects on rabbit fibrinogen [28,29].

In 2005, Howes *et al.*[30] isolated three metalloproteinases – EoVMP_1_, EoVMP_2_ and EoVMP_3_ – from the venom of *Echis ocellatus*. EoVMP_2_ and EoVMP_3_ provoked coagulation of human plasma and were considered procoagulation factors. They also led to disturbances in fibrin formation and caused systemic bleeding. All the three metalloproteinases were able to activate prothrombin and to convert it into different fractions [30].

Coagulation factors such as subfractions F_1_A and F_1_B – which we successfully purified, determined their molecular weight and completely identified in our later studies – may be used as important tools in laboratorial analysis, particularly related to liver diseases. Besides FXa, these enzymes act independently, eliminating the use of any cofactors, including FV, on carboxilated or nonocarboxilated prothrombin. Even in the presence of a FV disorder, the amount of prothrombin in patient blood may be measured using these enzymes [26,29].

In the hemostatic system, precise control of blood coagulation is mandatory for life. *Echis carinatus* venom is a rich source of compounds that influence several processes that occur in this system of prey organism. Some of these molecules may bring benefits to human health. For example, cardiac arrest, arterial obstruction and other cardiovascular and cerebral diseases are important causes of mortality throughout the globe. Atherosclerosis plays a major role in the pathophysiology of these diseases. Since blood clots consist of platelets and fibrin, treatment strategies have been developed based on coagulation, fibrinolysis and platelet functions.

Ec venom compounds may be used as medicines to treat thromboembolic disorders. Prothrombin-like enzymes used in defibrillation are part of the thrombolytic treatment (clotting decomposition). These enzymes break down fibrinogen, yet they do not activate FXIII. Broken down fibrinogen peptides are somehow similar to fibrin without transverse links which are quickly removed from the blood circulation. Once fibrinogen is removed from the blood, its viscosity will decrease and the blood circulation will be optimized. Some of Ec venom compounds may also be purified and used as procoagulant medicines. That is, some Ec venom proteins act on blood coagulation and possess at the beginning a thrombin-like activity followed by a thromboplastin-like activity. The first enzyme provokes fibrinogen coagulation by breaking down fibrinopeptide A, whereas the second activates FX [31].

These proteins accelerate platelet aggregation and, consequently, shorten coagulation time and reduce damage to blood, a property used to prevent and treat hemorrhage [2]. The lethal effects of snake venoms on different body systems have led to extensive studies on types of snake venom; differentiation and isolation of venoms into various fractions and further investigation of their biochemical or pathophysiological effects.

Enzymes obtained from snake venoms have been used to treat several diseases. Therefore, efforts to isolate fractions, purify different factors, analyze venom enzymes and toxicity properties have become more common. However, only a few actions have been taken so far in this regard in Iran. The Venomous Animal Unit of Razi Vaccine and Serum Research Institute is the main supplier of antivenom in the country. The scarce research has not only incited actions in this field, but also encouraged efforts to isolate *Echis carinatus* crude venom into different fractions and to study their enzymatic and toxic properties. This snake has been selected because of its massive presence in Iran and high incidence of snakebites. This research was conducted in cooperation with the Venomous Animal Unit of Razi Vaccine and Serum Research Institute in the hope that other scientists may not ignore this valuable source of biological compounds and may conduct further studies on this issue.

## Conclusions

The present study analyzed the venom of *Echis carinatus* snake with regard to coagulation activities. Its coagulation proteins were isolated and evaluated using chromatographic methods. Ec, a native species in Iran, has been killed by people for a long time because of its bites. Now, it may be used as a rich source of proteins that may be employed in the pharmaceutical industry.

## Ethics committee approval

The present study was approved by the Ethics Committee of Razi Vaccine and Serum Research Institute, Karaj, Iran.

## Competing interests

The authors declare no conflicts of interests.

## Authors’ contributions

All authors read and approved the final manuscript.

## Financial source

The Razi Vaccine and Serum Research Institute, Karaj, Iran, provided the financial grants.
